# On Toxic Neutralisations

**Published:** 1894-11-03

**Authors:** Benjamin Ward Richardson


### On Toxic Neutralisations.

By Sir Benjamin Ward Richardson, M.D., F.R.S.

### III.—PHYSIOLOGICAL MODIFICATION, OR CATALYSIS.

It will be seen from the final paragraph of my last paper that experiment was not extremely satisfactory, as indicating that the chemical reconstitution of a chemical substance in the body could be accepted as reliable. But I intimated that there were certain physiological modifications which were extremely important in the consideration of the action of various medicinal substances.

We have always to consider, in dealing with medicinal bodies, the changes we are about to induce by modification of physiological function. We may look on this point altogether apart from chemical reconstitution. It may happen that chemical reconstitution takes place, and that under the change new physiological developments are developed; but it does not seem necessary that, in order to produce the most distinctive effects there should be anything more than a physiological modification. Indeed, in a large number of results which occur from administration there is no actual change in the substance introduced, I mean change of the chemical order, while there is the most marked change in respect of physiological phenomena. In plain words, a substance may enter the body, may lead to the most distinctive effects or phenomena, and may be left in or pass out of the body without undergoing chemical alteration. To use a common example, chloroform may be adduced. We all understand what the phenomena produced by this substance are. We also understand that, in a general sense, the mode of introduction does not largely modify phenomena. We may administer it by the lungs so that a small portion of it being introduced into the pulmonary vesicles may pass into the blood by those channels. We may inject it subcutaneously and get absorption in that manner. We may administer it by the mouth, and let it pass into the circulation by the alimentary system; but in all instances it will, when it enters the circulation, produce virtually the same conditions. If into the blood a certain measure calculated by Snow and myself at eighteen minims gain access, then phenomena are manifested of the most marked character. They include the destruction of consciousness; the destruction of sensibility; sometimes reflexes that cause vomiting; a certain depression of temperature; and, occasionally, a kind of delirium. Nothing can be more extreme, in fact, than the modification of physiological function that takes place. All systems of the body are affected by the modification, and yet we have no reason to believe that the substance chloroform undergoes any chemical decomposition within the body. It is changed, most probably, in its mere physical characters. It is raised into the vaporous state under the heat of the body, and in the vaporous state it must of necessity be widely and intimately diffused through the vascular and nervous systems, inducing, temporarily, an actual physical perversion, or what may be called a molecular extension of these systems; but, as far as I could ever discover, the chloroform itself undergoes no decomposition. When recovery takes place it exhales as chloroform from the excreting organs. If, after death from the action of chloroform, the tissues be examined chemically, there is no evidence of the presence of any new thing except chloroform; in fact, throughout it remains a simple disturbing element, disturbing by its actual presence—a foreign substance running its course through the body, and nothing more. There are many other substances which can be treated of precisely in a similar manner. They act as foreign substances without undergoing modification of chemical form. The hydrides, to which I have already called attention, belong to this family of physiological workers, and because they are insoluble in the blood they act as most decisive mechanical obstructions to the course of the blood, almost as if they were really solid bodies circulating with the blood, in an insufficient form of sub-division to pass through the capillaries under compression, or insufficiently divided to make their way through the capillary tubes as corpuscles do. I found, for instance, that when death takes place from the effects of a hydride introduced into the circulation that the mode of death is as by a plug, and that actually the globule of the hydride can be found in the blood in the free state. But there is no proof whatever that the hydride itself is chemically altered. It is a highly combustible substance, as we know; burns with an intense flame; is, indeed, explosive. But there is not sufficient heat in the system nor process of metamorphosis to lead to its combustion. The amyl hydride is probably the most easily of explosive fluids under ordinary pressure, yet it undergoes no combustion in the body. If it did, the blood would be set into fever when mixed with it in intimate combination. It produces certain actions nevertheless. It causes stupor; it causes insensibility; it excites to vomiting, and it very easily leads to death. Yet its properties are all physiological, not chemical. Doubtless it, like chloroform, may, by its presence in the lung, interfere with chemical combination. That is most likely, for it does so, as I found, out of the body, on substances that oxydise in the air, or in oxygen; but this again is only a physiological effect interfering with the effect of other chemical agents, and not acting by destruction of its own chemical constitution.

The same rule applies to more distinctively solid substances. It applies to such a body as strychnine. Everything in the way of evidence goes to prove that strychnine, diffused through the tissues, undergoes no change. It is diffused widely through the tissues, and, so diffused, it causes the most determinate effects; it leads the muscles to assume tetanic contractions attended with intense pain. It does not touch, apparently, the sensory system at all, but it strongly excites the motor nerves, or it acts directly on the muscular fibre, we know not correctly which, but it undergoes no change in itself. It remains in the body unaffected in composition, and it even resists in the dead body the process of decomposition of tissue. It happened once, in the course of a great trial, that the remains of a dog that had been poisoned by strychnine were extracted from the earth after burial for three years, and were examined by another observer and myself for strychnine. We found in this debris the evidence of strychnine, as if the animal had not been dead an hour. The strychnine had produced all its fatal effects by its presence, remaining itself intact. It would be possible to extend these comparisons to atropina, to canabina, and to the majority of the organic substances which we employ in medical practice, when they are stripped of their extraneous surroundings; but the illustrations given are all sufficient to make obvious what may be called physiological modification, or more properly, perhaps, physiological catalysis.

